# Basal Cell Carcinoma With Matrical Differentiation Case Report and Review of Management

**DOI:** 10.7759/cureus.101253

**Published:** 2026-01-10

**Authors:** Ryan Wealther, Tramondranique Hawkins, Dora R Goldstein, Keith Wisniewski, Floyd A Pirtle, Justin Raman, Kritin K Verma, Michelle Tarbox

**Affiliations:** 1 Dermatology, Texas Tech University Health Sciences Center, Lubbock, USA; 2 Medicine, Texas Tech University Health Sciences Center, Lubbock, USA; 3 Medicine, Texas Tech University Health Sciences Center El Paso Paul L. Foster School of Medicine, El Paso, USA

**Keywords:** basal cell carcinoma, matrical differentiation, mohs micrographic surgery, pilomatrixoma, shadow cells

## Abstract

Basal cell carcinoma with matrical differentiation (BCCMD) is a rare subtype of basal cell carcinoma (BCC), with only about 43 cases described in the literature. It is characterized by typical BCC morphology, including basaloid cell aggregates with peripheral palisading, along with areas of matrical differentiation containing shadow cells, trichohyalin granules, and corneocytes. Immunohistochemically, the basaloid component expresses BerEP4, while the matrical component often shows diminished BerEP4 and positive β-catenin and epithelial membrane antigen staining. BerEP4 represents an immunohistochemical marker that highlights basaloid cells and is commonly used to aid in identifying basal cell carcinoma and distinguishing it from other skin tumors. A 79-year-old man presented with a new 9-mm pearly papule on the right forehead, which was diagnosed as BCCMD following histopathologic and immunohistochemical evaluation. The lesion was successfully treated with one stage of Mohs micrographic surgery, and the patient remained disease-free after nine months of follow-up. Due to overlapping histologic features with other follicular neoplasms, diagnosis is critical, and management strategies are not well established. While most cases have been treated with standard excision, Mohs surgery may offer superior margin control, especially for tumors in cosmetically sensitive regions. Rare reports of metastasis suggest a potentially more aggressive clinical course than typical BCC. Comprehensive excision and long-term surveillance are recommended to optimize outcomes and further characterize this rare variant.

## Introduction

Basal cell carcinoma (BCC) is the most common skin cancer, accounting for over 80% of non-melanoma cases [1]. Five main histological subtypes are recognized: nodular, superficial, micronodular, infiltrating, and morpheaform, although other uncommon subtypes exist [2]. Basal cell carcinoma with matrical differentiation (BCCMD) is an uncommon subtype, with only about 43 occurrences reported in the literature, mostly as isolated cases [3].

BCCMD is distinguished by the combination of typical BCC characteristics, such as aggregates of basaloid cells with peripheral palisading as well as areas of matrical differentiation, which involves the production of shadow cells, trichohyalin granules, and corneocytes within the tumor [2-4]. These features are normally seen in the hair follicle’s growth zone, suggesting that this cancer may arise from cells capable of forming hair-like structures [2,3]. Immunohistochemistry reveals that the BCC component frequently expresses BerEP4, while the matrical portions show diminished or negative BerEP4 staining and positive staining for β-catenin and epithelial membrane antigen [2,4].

Clinically, BCCMD is important because its histopathologic overlap with other follicular neoplasms may lead to misdiagnosis. Additionally, rare reports of aggressive behavior and metastasis raise concern for a potentially more malignant course than conventional BCC. Awareness of this entity is therefore essential to ensure accurate diagnosis, appropriate surgical management, and long-term follow-up.

Surgical treatment remains the standard treatment for BCC, with Mohs micrographic surgery providing the greatest cure rates [3]. However, because BCCMD is uncommon, the best way to handle therapeutic options has not yet been determined.

## Case presentation

**Figure 1:**
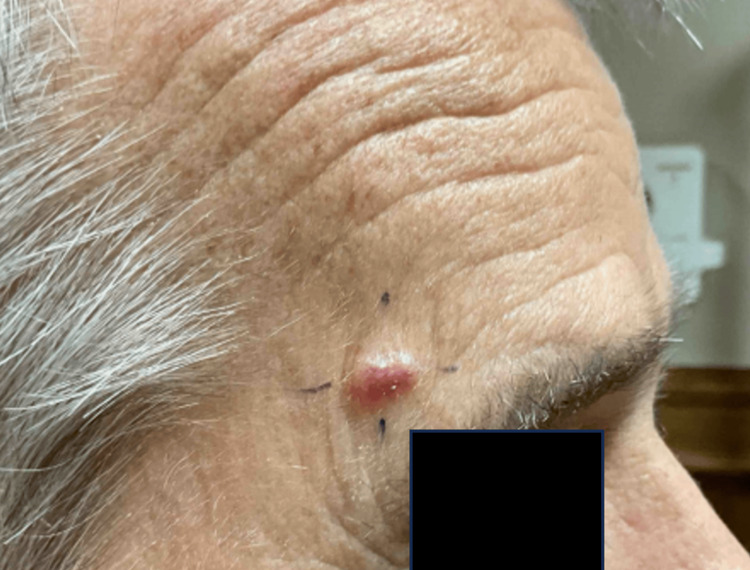
9-mm ovoid, pearly papule on the right forehead. The patient noted the lesion had been present for one month and was growing.

A 79-year-old man with a history of basosquamous cell carcinoma and squamous cell carcinoma reported a new lesion on his right forehead that he had discovered one month prior (Figure 1). Physical examination revealed a 9-mm, ovoid, pearly papule on his right forehead. The lesion was shave-biopsied and sent for pathological examination.

**Figure 2:**
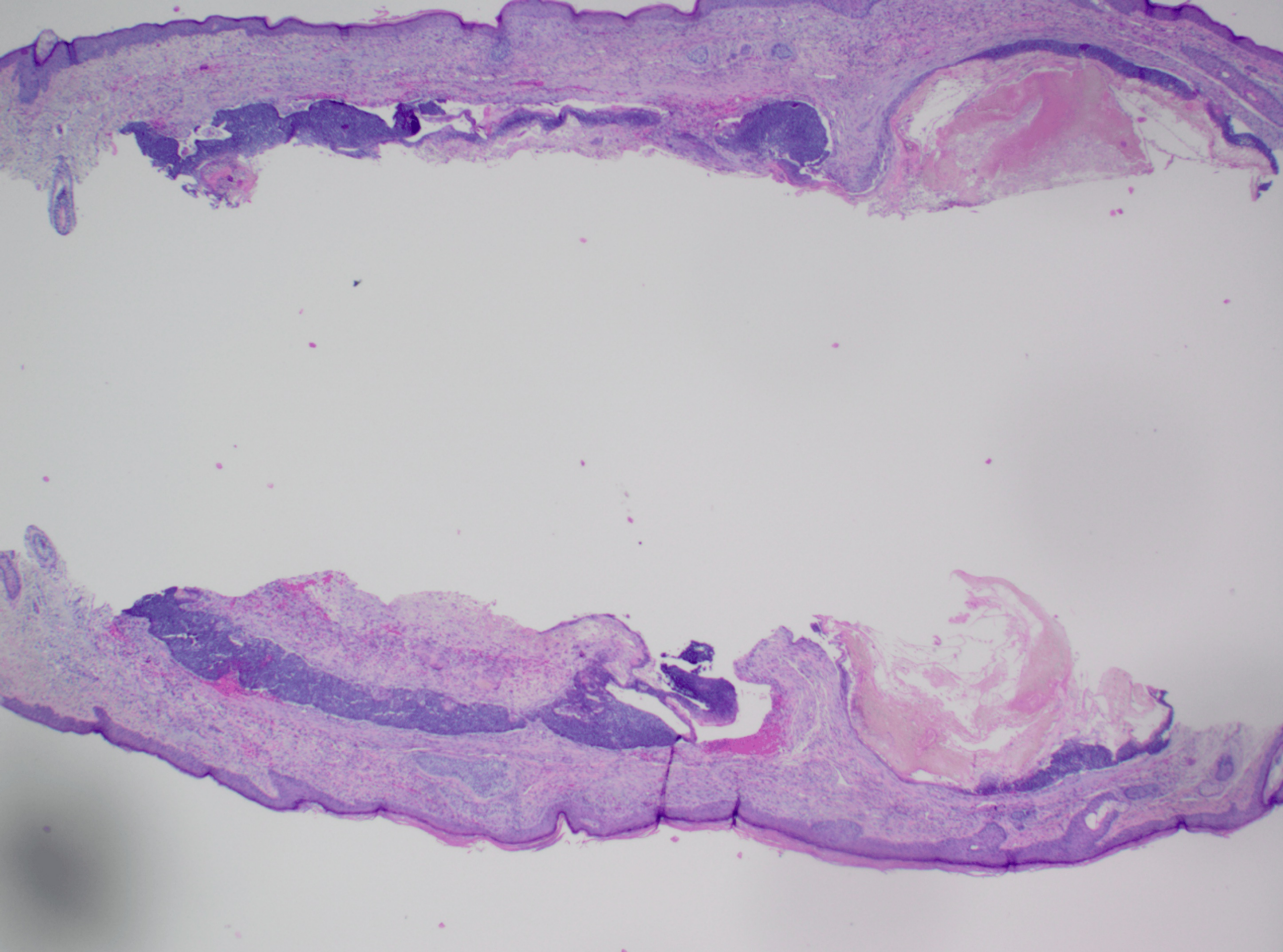
Hematoxylin and eosin staining at 40x magnification. Nodular aggregates of basaloid cells with peripheral palisading of nuclei and an increased cytoplasmic ratio in the dermis. Deeper in the dermis, cystic spaces lined by basaloid cells with aggregates of shadow cells within the intratumor spaces are present.

**Figure 3:**
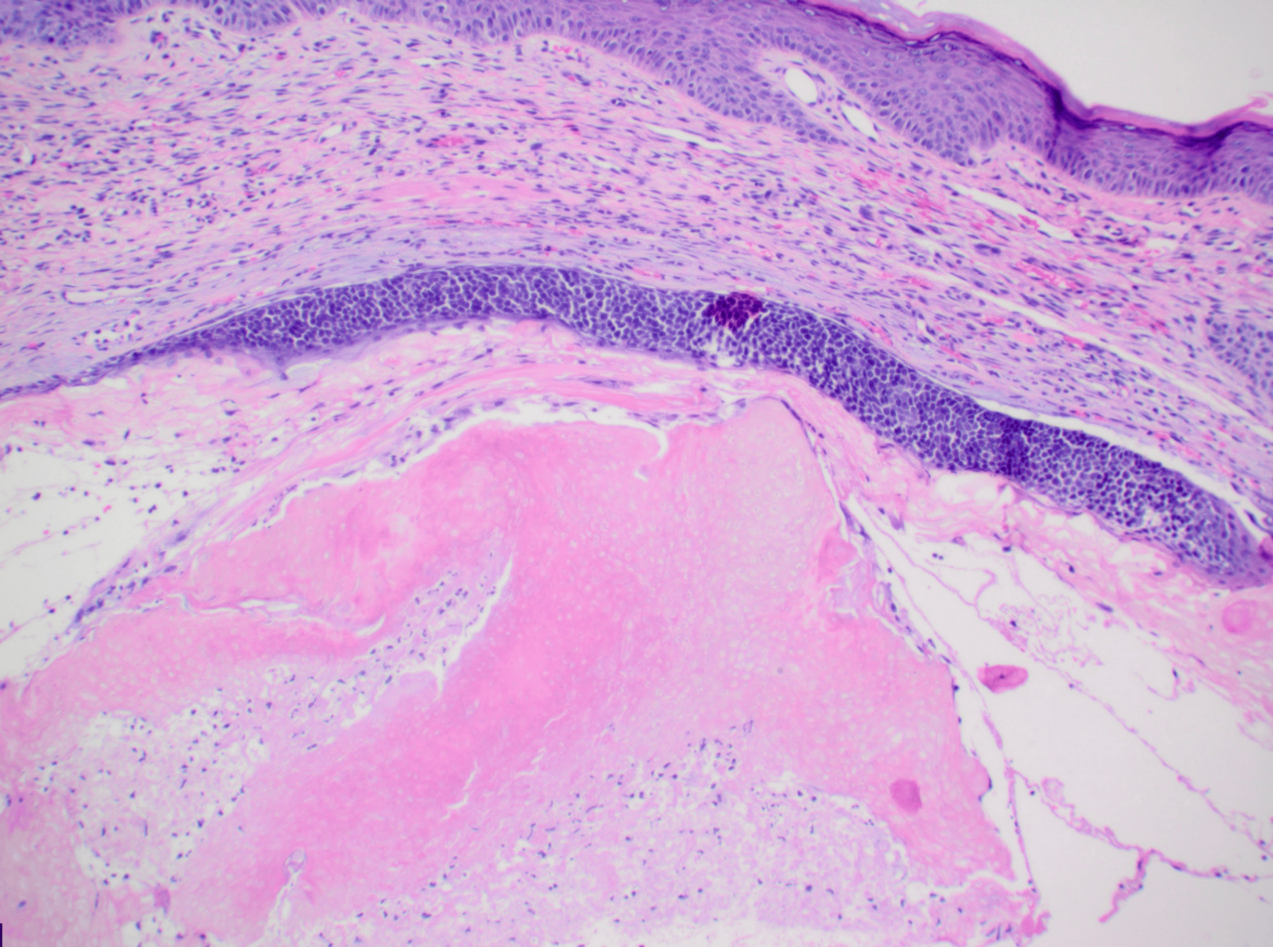
Hematoxylin and eosin staining at 100x magnification. Aggregates of pink ghost cells are seen clearly in the intratumor cystic space, which is lined by basaloid cells.

**Figure 4:**
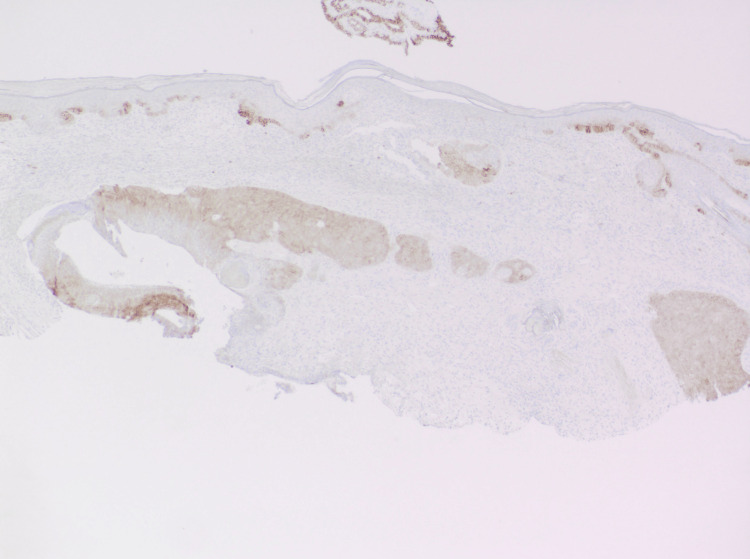
Ber-EP4 highlights the dermal nodules of basaloid cells along with the basaloid cells lining the cystic space at 40x magnification.

On dermatopathology, sections revealed a dermal tumor with nodular aggregates of basaloid cells with peripheral palisading of nuclei and an increased nuclear to cytoplasmic ratio, budding from the undersurface of the epidermis, and extending into the dermis. Clear cell change was noted, and there was a focal retraction artifact. The deep dermis was noted to have a cystic space lined by basaloid cells with retraction artifact and aggregates of shadow cells within intratumor cystic spaces. Ber-EP4 highlighted the dermal nodules of basaloid cells along with the basaloid cells lining the cystic space (Figures 2-4). A final diagnosis of basal cell carcinoma with pilomatrical differentiation was made. The patient was referred for Mohs surgery, and the lesion was cleared after one stage. The patient remains free of recurrence and metastasis nine months after his initial biopsy.

## Discussion

BCCMD is an uncommon subtype of basal cell carcinoma, with limited cases reported in the literature [5,6]. Treatment techniques, however, are largely similar to those used for conventional BCC, with some modifications to account for its distinct histological characteristics [2].

While Mohs micrographic surgery (MMS) is widely regarded as the gold standard for high-risk BCCs, the best therapeutic option for BCCMD remains unknown [7]. The majority of reported cases of BCCMD were treated with routine surgical excision [8]. There are only a few reported cases of BCCMD that were treated with MMS [4]. MMS has the advantage of providing comprehensive margin assessment intraoperatively, which may be especially useful for malignancies in cosmetically sensitive locations or with ill-defined margins [9].

While BCC has limited metastatic potential in general, metastasis in BCCMD has been reported on rare occasions [8]. Two (4.65%) of the 43 reported cases of BCCMD were metastatic, suggesting that BCCMD may have a higher metastatic potential than typical BCC [5,8]. The potential for more aggressive behavior in BCCMD may be explained by its underlying biological features. Matrical differentiation is closely associated with activation of the Wnt/β-catenin signaling pathway, which plays a critical role in hair follicle development, stem cell maintenance, and cellular proliferation. Aberrant nuclear accumulation of β-catenin has been implicated in pilomatrix carcinoma and other hair matrix tumors, which demonstrate higher rates of local recurrence and metastasis than conventional BCC. Therefore, BCCMD may represent an intermediate biological entity between conventional BCC and pilomatrix carcinoma [4,8].

Patients diagnosed with BCCMD should be closely monitored as they may grow rapidly [8,10]. Imaging and skin examinations may be used to monitor for local recurrence or metastatic disease, particularly in instances with high-risk characteristics: large tumor size, deep penetration, or perineural involvement [8].

BCCMD presents management issues due to limited clinical expertise [8]. While surgical excision remains the primary therapeutic option, the decision between regular excision and MMS should be based on the particular tumor features and patient considerations [4,7]. The possibility of enhanced aggressiveness, including the rare occurrence of metastasis, emphasizes the significance of full tumor excision and continuous monitoring [2,8]. More studies and long-term follow-ups on BCCMD cases are required to understand their biological behavior better and optimize therapeutic options.

## Conclusions

Basal cell carcinoma with matrical differentiation (BCCMD) remains a rare variant of BCC, and the limited representation in literature presents barriers for establishing standardized diagnostic and therapeutic guidelines. Recognition of the characteristic immunohistochemical and histopathologic features is essential for accurate diagnosis, particularly given its overlap with differing follicular tumors. Although BCCMD generally behaves similarly to conventional BCC, isolated reports of metastasis and more aggressive clinical courses suggest that matrical differentiation can confer an increased biologic potential in some cases. These observations highlight the importance of distinguishing BCCMD from other adnexal neoplasms that may share overlapping features. Continued awareness and careful diagnostic evaluation can help ensure timely and accurate identification of this uncommon entity. 

Surgical management remains the primary treatment modality, with Mohs micrographic surgery offering maximal margin control when lesions arise in cosmetically sensitive or high-risk regions. The present case supports that complete excision can achieve favorable outcomes, as demonstrated by the patient's disease-free status nine months following surgery. Nevertheless, the rarity of BCCMD and the potential of heightened aggressiveness underscore the importance of meticulous tumor removal and ongoing clinical surveillance. In practice, follow-up with full skin examinations at six to 12-month intervals for the first two to three years may be reasonable, particularly in the absence of established guidelines. Continued reporting of BCCMD cases is essential to refine the understanding of its behavior and to inform future management recommendations. These considerations are particularly relevant in cases demonstrating deep invasion, perineural involvement, or ill-defined tumor borders. Ongoing accumulation of well-documented cases will be crucial for clarifying the natural history of BCCMD and guiding evidence-based management strategies going forward. 
